# Will Avatars Offer Answers?

**Published:** 2007-06-15

**Authors:** Lynne S. Wilcox

Though we often scold ourselves for having limited perceptions, public health professionals understand the relationship between community wellness and personal health better than most. We recognized this as we reviewed the recommendations of the National Expert Panel on Community Health Promotion (1). *Preventing Chronic Disease* and other public health journals have published articles that address the essence of these recommendations, including the topics of community-level surveillance (2-4); community-based participatory research (5,6); wellness and community, including mental health and complementary and alternative medicine (7-11); and training and capacity building (12-16). Certainly no discussion of public health action is complete without examining funding approaches such as those the expert panel described (17-19). We thank Dr Leandris Liburd and Ms Amanda Navarro of the Division of Adult and Community Health, National Center for Chronic Disease Prevention and Health Promotion, Centers for Disease Control and Prevention, for serving as guest editors of this issue.

The expert panel also recommended the establishment of online communities for sharing information and promoting dialogue on evidenced-based approaches to community health — a virtual community that "combines the best of Wikipedia, Google, MySpace, [and] Meetup (1)." More specific panel recommendations for this virtual community include encouraging participants to share their experiences and increasing opportunities for them to contribute information about communications tools, methods of collecting data on community indicators, training, and evaluation. Crespo elaborates on this concept in his discussion of new opportunities in online communication (20).

Here is an additional source of inspiration for the virtual community: *SimCity*. *SimCity* is a computer game that allows players to create infrastructure models of entire cities and to simulate events such as disasters and terrorism. It was first released in the 1980s and has since become one of the most popular computer games of all time (21). While *SimCity* was built for entertainment, a developing field known as "serious games" intends to offer education, training, and participant interaction and to inform policy-making (22).

The modeling of health outcomes based on demographics, disease information, and risk factors has been used for years in public health science. But these models are usually not transparent; interpreting them typically requires extensive understanding of biostatistics and subject content, and their users cannot interact with them. Serious games, in contrast, operate according to the same types of sophisticated underlying models but have a user-friendly interface that allows players to discover for themselves the consequences of different policy choices. The Social Impact Games Web site lists multiple health, wellness, and policy games (23), including *EpiSims*, created by the Los Alamos National Laboratory, a modeling game that examines the theoretical spread of infectious disease in a community using data about the disease, the transmission from host to host, the social contact networks, and other relevant aspects of the setting. The model allows adjustment of parameters, such as age distribution in the community, to examine differences in epidemic outcome (24).

Appealing as it may be, a serious game is only as valuable as its assumptions. The virtual community recommended by the expert panel could be an important source of data for creating robust assumptions, many of which would address real-world dynamics. Use of current resources such as the *Community Guide to Preventive Services* (25) would provide evidence-based examples of effective interventions in community settings that could inform the development of sound models. Students could use this modeling to improve their understanding of how aspects of the community, such as its built environment, affect the wellness of its population. Indeed, given the average age of gamers, younger professionals may be the people most likely to design and use these computerized learning environments effectively.

Many games allow the selection of "avatars," or characters that represent the player in the game, somewhat like the tokens used in children's board games to represent each player's progress. Each avatar is unique and interactive, and each player may select or change avatars to experience the game from different perspectives. The United Nations World Food Programme provides an online serious game that allows players to select an avatar child or adult living in a refugee camp in Darfur. The avatar might be responsible for bringing water into camp, building shelter, or obtaining food, and must avoid wartime threats to complete the task (26).

In a serious game for community settings, avatars could change from one game to the next to enable players to understand the experience of a mayor, a public health nurse, an academician, or other community members. Bringing multiple partners into the game setting over time could facilitate the adoption of revisions based on the on-the-ground experiences of individual communities and enable better understanding of social factors. Implementation of other recommendations by the expert panel could be informed by the results of these simulations.

Such games may be helpful to policy-makers because prospective models are useful in determining where resources are best applied (27). Of course, these games do not offer crystal balls. But examining results under different assumptions will encourage discussion among key decision-makers and may allow more rapid recognition of emerging factors that could affect health outcomes. The advantage of investigating these factors in a game setting is that citizens need not be modeling experts to appreciate which results are meaningful. Developing such games will require a partnership of modelers and community observers, as well as sponsors of the research and the creation of design elements. As we have seen in the electronic world, technologic development is an iterative process. A hypothetical *Public Health Game 1.0* will be replaced by version 2.0 as community-based practice and research provide more answers.

## References

[1] Navarro A, Voetsch K, Liburd L, Bezold C, Rhea M (2006). Recommendations for future efforts in community health promotion: report of the National Expert Panel on Community Health Promotion..

[2] MacDonald G, Garcia D, Zaza S, Schooley M, Compton D, Bryant T (2006). Steps to a HealthierUS Cooperative Agreement Program: foundational elements for program evaluation planning, implementation, and use of findings. Prev Chronic Dis.

[3] Roberts EM, English PB, Wong M, Wolff C, Valdez S, Van den Eeden SK (2006). Progress in pediatric asthma surveillance II: geospatial patterns of asthma in Alameda County, California. Prev Chronic Dis.

[4] Pluto DM, Phillips MM, Matson-Koffman D, Shepard DM, Raczynski JM, Brownstein JN (2004). Policy and Environmental Indicators for Heart Disease and Stroke Prevention: Data Sources in Two States. Prev Chronic Dis.

[5] Harris JR, Brown PK, Coughlin S, Fernandez ME, Hebert JR, Kerner J (2005). The Cancer Prevention and Control Research Network. Prev Chronic Dis.

[6] Kelley MA, Baldyga W, Barajas F, Rodriguez-Sanchez M (2005). Capturing Change in a Community–University Partnership: The ¡SíSe Puede! Project. Prev Chronic Dis.

[7] Polacsek M, O'Brien LM, Lagasse W, Hammar N (2006). Move & Improve: a worksite wellness program in Maine. Prev Chronic Dis.

[8] McKeever C, Koroloff N, Faddis C (2006). The African American Wellness Village in Portland, Ore. Prev Chronic Dis.

[9] Stewart AL, Gillis D, Grossman M, Castrillo M, Pruitt L, McLellan B (2006). Diffusing a research-based physical activity promotion program for seniors into diverse communities: CHAMPS III. Prev Chronic Dis.

[10] Colton CW, Manderscheid RW (2006). Congruencies in increased mortality rates, years of potential life lost, and causes of death among public mental health clients in eight states. Prev Chronic Dis.

[11] Dente JM, Herman CJ, Allen P, Hunt WC (2006). Ethnic differences in the use of complementary and alternative therapies among adults with osteoarthritis. Prev Chronic Dis.

[12] Yee SL, Williams-Piehota P, Sorensen A, Roussel A, Hersey J, Hamre R (2006). The Nutrition and Physical Activity Program to Prevent Obesity and Other Chronic Diseases: monitoring progress in funded states. Prev Chronic Dis.

[13] Wolbeck Minke S, Smith C, Plotnikoff RC, Khalema E, Raine K (2006). The evolution of integrated chronic disease prevention in Alberta, Canada. Prev Chronic Dis.

[14] Plescia M, Young S, Ritzman RL (2005). Statewide community-based health promotion: a North Carolina model to build local capacity for chronic disease prevention. Prev Chronic Dis.

[15] Franks AL, Brownson RC, Bryant C, Brown KM, Hooker SP, Pluto DM (2005). Prevention Research Centers: contributions to updating the public health workforce through training. Prev Chronic Dis.

[16] Thacker SB (2005). How do we ensure the quality of the public health workforce?. Prev Chronic Dis.

[17] Bobbitt-Cooke M (2005). Energizing community health improvement: the promise of microgrants. Prev Chronic Dis.

[18] Butterfoss FD, Dunět DO (2005). State Plan Index: a tool for assessing the quality of state public health plans. Prev Chronic Dis.

[19] Hedworth AB, Smith CS (2006). The Great Lakes Regional Stroke Network experience. Prev Chronic Dis.

[20] Crespo R (2007). Virtual community health promotion. Prev Chronic Dis.

[21] Inside scoop: the history of SimCity.

[22] Serious games initiative.

[23] Social impact games.

[24] Goetz T (2006). The battle to stop bird flu. Wired.

[25] The community guide to preventive services.

[26] Food force.

[27] Milstein B, Jones A, Homer J, Murphy D, Essien J, Seville D (2007). Charting plausible futures for diabetes prevalence in the United States: A role for systems dynamic simulation modeling. Prev Chronic Dis.

